# Draft Genome Sequence of the *Xanthomonas bromi* Type Strain LMG 947

**DOI:** 10.1128/genomeA.00961-16

**Published:** 2016-09-08

**Authors:** Lena Hersemann, Daniel Wibberg, Jochen Blom, Franco Widmer, Roland Kölliker

**Affiliations:** a Molecular Ecology, Institute for Sustainability Sciences, Agroscope, Zurich, Switzerland; b Center for Biotechnology, Bielefeld University, Bielefeld, Germany; c Bioinformatics and Systems Biology, Justus-Liebig-University Giessen, Giessen, Germany

## Abstract

Here, we report the draft genome sequence of the *Xanthomonas bromi* type strain LMG 947, an important pathogen of bromegrasses (*Bromus* spp.). Comparative analysis with other *Xanthomonas* spp. that are pathogenic on forage grasses will assist the analysis of host-plant adaptation at the genome level.

## GENOME ANNOUNCEMENT

Two *Xanthomonas* spp. (*X. translucens* and *X. bromi*) are known as grassland pathogens (1). While both species cause bacterial wilt on forage grasses, DNA-DNA hybridization assays and analysis of both protein and fatty acid methyl ester profiles have revealed distinct interspecies differences (2). Phylogenetic analysis of *Xanthomonas* spp. using multilocus sequence analysis of various housekeeping genes (i.e., *gyrB*, *rpo*D, *dnaK*, and *fyuA*) divided the analyzed 119 strains into two major groups (3). These two groups have been found to separate the two forage grass–affecting *Xanthomonas* spp. and thus suggest a high genetic distance for both *X. bromi* and *X. translucens*. Several studies on the pathogenesis (4, 5) and genome mining of virulence-related traits (6) have contributed to an understanding of the underlying mechanisms of bacterial wilt caused by *X. translucens* pathovars; however, no genome information was available for *X. bromi*. Reports on bacterial wilt caused by *X. bromi* indicate a wide distribution of this pathogen throughout New Zealand and France among a variety of different *Bromus* spp. (7, 8). The type strain LMG 947 (BCCM/LMG culture collection of the Laboratory of Microbiology, Ghent University, Belgium) was isolated in 1980 in France from the species *Bromus carinatus*, which shows high susceptibility for bacterial wilt caused by *X. bromi* (2, 7).

In this study, the *X. bromi* LMG 947 genome was sequenced to get the first insights into its gene content. The paired-end (2 × 300-bp) sequencing run on the Illumina MiSeq platform (Illumina Inc, San Diego, California, USA) resulted in 4,880,586 reads yielding approximately 1.4 Gb of sequence data. A *de novo* assembly of the obtained Illumina sequence reads applying Newbler Assembly version 2.8 software (Roche, Basel, Switzerland) resulted in 84 scaffolds and 134 contigs. The software platform GenDB (9) was applied to annotate the *X. bromi* LMG 947 draft genome. The *X. bromi* LMG 947 draft genome features a 4,843,951-bp chromosome harboring an average GC content of 64.05%. A total of 4,190 protein coding sequences, 53 tRNAs, and three rRNAs were predicted. *Xanthomonas* spp. share a variety of common virulence-related traits like the type III secretion system (T3SS) encoded by the hypersensitive response and pathogenicity gene cluster (10), as well as type III secreted effector proteins (11). The LMG 947 genome was found to harbor a canonical T3SS (12), as well as 28 putative type III effector proteins identified by BLASTp analysis against the effectors listed in the online database of *Xanthomonas* effector proteins (http://xanthomonas.org/t3e.html). A homology search revealed that the putative effector proteins belong to 22 different effector classes (i.e., AvrBs2, XopA, XopAD, XopAE, XopAF, XopAG, XopAH, XopAJ, XopAM, XopE, XopF, XopH, XopI, XopK, XopL, XopN, XopP, XopQ, XopR, XopV, XopX, and XopZ). Moreover, the genome data of LMG 947 indicate the presence of a transcription activator–like effector class AvrBs3 homologue (13). With respect to the high genetic and phenotypic differences between forage grass–affecting *Xanthomonas* spp., the *X. bromi* type strain LMG 947 will complement genomic information of forage grass–affecting *X. translucens* pathovars and may facilitate the identification of conserved traits linked to host adaptation.

### Accession number(s).

This whole-genome shotgun project has been deposited in DDBJ/ENA/GenBank under the accession numbers FLTX01000001 to FLTX01000134. The version described in this paper is the first version, FLTX01000000.
